# Estimated Association of Construction Work With Risks of COVID-19 Infection and Hospitalization in Texas

**DOI:** 10.1001/jamanetworkopen.2020.26373

**Published:** 2020-10-29

**Authors:** Remy F. Pasco, Spencer J. Fox, S. Claiborne Johnston, Michael Pignone, Lauren Ancel Meyers

**Affiliations:** 1 Department of Integrative Biology, The University of Texas at Austin; 2 Dell Medical School, The University of Texas at Austin; 3 Santa Fe Institute, Santa Fe, New Mexico

## Abstract

**Importance:**

Policy makers have relaxed restrictions for certain nonessential industries, including construction, jeopardizing the effectiveness of social distancing measures and putting already at-risk populations at greater risk of coronavirus disease 2019 (COVID-19) infection. In Texas, Latinx populations are overly represented among construction workers, and thus have elevated rates of exposure that are compounded by prevalent high-risk comorbidities and lack of access to health care.

**Objective:**

To assess the association between construction work during the COVID-19 pandemic and hospitalization rates for construction workers and the surrounding community.

**Design, Setting, and Participants:**

This decision analytical model used a mathematical model of COVID-19 transmission, stratified by age and risk group, with construction workers modeled explicitly. The model was based on residents of the Austin–Round Rock metropolitan statistical area, with a population of 2.17 million. Based on 500 stochastic simulations for each of 15 scenarios that varied the size of the construction workforce and level of worksite transmission risk, the association between continued construction work and hospitalizations was estimated and then compared with anonymized line-list hospitalization data from central Texas through August 20, 2020.

**Exposures:**

Social distancing interventions, size of construction workforce, and level of disease transmission at construction worksites.

**Main Outcomes and Measures:**

For each scenario, the total number of COVID-19 hospitalizations and the relative risk of hospitalization among construction workers was projected and then compared with relative risks estimated from reported hospitalization data.

**Results:**

Allowing unrestricted construction work was associated with an increase of COVID-19 hospitalization rates through mid-August 2020 from 0.38 per 1000 residents to 1.5 per 1000 residents and from 0.22 per 1000 construction workers to 9.3 per 1000 construction workers. This increased risk was estimated to be offset by safety measures (such as thorough cleaning of equipment between uses, wearing of protective equipment, limits on the number of workers at a worksite, and increased health surveillance) that were associated with a 50% decrease in transmission. The observed relative risk of hospitalization among construction workers compared with other occupational categories among adults aged 18 to 64 years was 4.9 (95% CI, 3.8-6.2).

**Conclusions and Relevance:**

The findings of this study suggest that unrestricted work in high-contact industries, such as construction, is associated with a higher level of community transmission, increased risks to at-risk workers, and larger health disparities among members of racial and ethnic minority groups.

## Key Points

**Question:**

Is construction work associated with increased community transmission of coronavirus disease 2019 (COVID-19) and disproportionate morbidity among construction workers in US cities?

**Findings:**

This decision analytical model of COVID-19 found that resuming construction work during shelter-in-place orders was associated with increased hospitalization risks in the construction workforce and increase transmission in the surrounding community. Based on COVID-19 hospitalization data through August 20, 2020, construction workers had a nearly 5-fold increased risk of hospitalization in central Texas compared with other occupational categories.

**Meaning:**

The findings of this study suggest that enacting workplace safety policies and providing paid sick leave could protect essential workers in high-contact industries and prevent further widening of disparities in COVID-19 morbidity and mortality.

## Teaser

This decision analytical model assesses the association between construction work during the coronavirus disease 2019 (COVID-19) pandemic and hospitalization rates for construction workers and the surrounding community.

## Introduction

During March 2020, cities across the United States enacted stay-at-home orders to combat the emergence of the coronavirus 2019 (COVID-19) pandemic. Essential industries, such as health care, transportation, energy, and food, were kept open, while nonessential industries, such as restaurants and entertainment, were largely prohibited. However, policy makers were divided on construction work. Boston, New York, and San Francisco severely restricted allowable projects.^1,2^ Other cities and states deemed commercial and home construction essential.^3^ Most of the nation’s 7.3 million construction workers remained employed throughout April and May of 2020, representing 4.5% of the labor workforce, ranging from 1.8% in the District of Columbia to 10.5% in Wyoming.^4^ The risk of viral transmission on construction worksites is amplified by the physical proximity required for many tasks.^5^ Latinx populations are overly represented among construction and essential industries,^6^ and thus have elevated rates of exposure that are compounded by prevalent high-risk comorbidities and lack of access to health care.^7^ These overlapping risks likely contribute to the disproportionate burden of COVID-19 infections and deaths reported within Latinx communities.^8^

On March 24, 2020, the city of Austin, Texas enacted a Stay Home–Work Safe order that limited construction work to projects designated as critical infrastructure. This excluded commercial and residential construction.^9^ A week later, the Texas governor overruled this restriction, declaring all construction work permissible statewide.^10^ At the time, the authors of this study conducted a risk assessment per a request from the mayor of Austin and judge of Travis County. It was determined that construction work might undermine the efficacy of the stay-home order, accelerating spread and amplifying risk in a workforce with overlapping risks.^11^ The early projections were corroborated by heightened hospitalization rates within the Austin construction workforce between March 13 and August 20, 2020.

In general, subgroups that engage in activities that increase their exposure to the virus are likely to experience a disproportionate burden of disease and, if socially connected to the surrounding community, cause a disproportionate degree of community spread. In this study, we quantified these risks for the construction industry in Austin, Texas, during the early months of the COVID-19 pandemic as a data-driven demonstration of this general phenomenon.

## Methods

A data-driven model of COVID-19 transmission was used to estimate the association between construction work and the effective reproduction number (*R_e_*) of the virus. The model captures age-specific high-risk proportions and contact rates between the general public and Austin’s approximately 50 000 construction workers. The projections hinge on the efficacy of measures to protect health and safety at worksites, such as health monitoring and wearing face masks.

The hospitalization data analyzed in this study were provided in deidentified files by the City of Austin. This study qualifies for an exemption of informed consent per the Common Rule with an approval by the institutional review board of The University of Texas at Austin currently pending. This analysis adheres to the Consolidated Health Economic Evaluation Reporting Standards (CHEERS) reporting guideline, where applicable.

### Transmission Model

The stochastic age-structured and risk-structured compartmental model used in this study describes the epidemiological transmission dynamics of severe acute respiratory syndrome coronavirus 2 (SARS-CoV-2) within and between 217 metropolitan areas in the United States. In each metropolitan area, the model includes 5 age groups,^12^ each with 3 risk groups (ie, low risk, high risk, and pregnant) derived from data on high-risk comorbidities available through the US Centers for Disease Control and Prevention (CDC) 500 Cities Project^13^ as well as on the local prevalence of HIV,^14^ morbid obesity,^15,16^ births,^17^ and abortions^18^ (eAppendix 1 in the Supplement). The transmission of the virus is represented by equations that track the movement of individuals among several disease states: susceptible, exposed, presymptomatic, asymptomatic, symptomatic, hospitalized, and recovered (eAppendix 2 in the Supplement).

For this analysis, the submodel for the Austin–Round Rock metropolitan statistical area (MSA) was modified to explicitly include Austin’s construction workforce. There are an estimated 50 000 construction workers in the Austin metropolitan area, representing more than 4% of the labor force,^19^ not accounting for undocumented workers. The model assumes that all construction workers are aged 18 to 49 years with the same high-risk proportion as in the general population in the base case.

**Figure 1.**
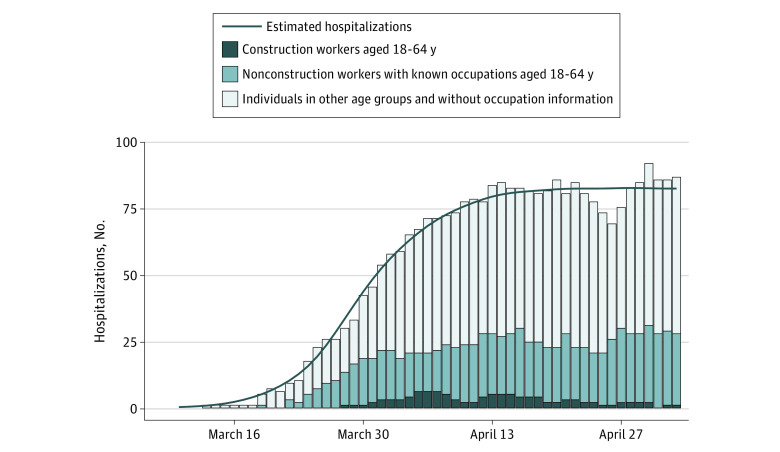
Reported and Projected Coronavirus Disease 2019 Hospitalizations in the Austin–Round Rock Metropolitan Area Reported hospitalizations (bars) from March 10 to May 3, 2020, compared with estimates (line) based on a susceptible-exposed-infected-recovered compartmental model that considers age-specific high-risk groups and contact rates for the general population of 2.17 million and a construction workforce of 50 000 workers.

The start date and transmission rate (β) of the model are calibrated simultaneously by statistical fitting to local hospitalization rates, finding the parameters that generated forecasts of the number of hospitalizations for the entire population that most closely matched the actual data by May 3 (Figure 1). The model assumes a constant transmission rate before the declaration of the stay-at-home order on March 24, 2020, and a constant reduction in contacts after the order that directly reduced the transmission rate. The details of the fitting method are provided in eAppendix 2 in the Supplement. Using the transmission rate, the implied basic reproductive number and doubling time prior to intervention are computed using a next-generation matrix.^20^

Simulations begin with 5 presymptomatic cases in individuals aged 18 to 49 years on February 29, 2020, and update at 2.4-hour intervals. For each combination of construction workforce size (0% to 100% in 25% increments) and relative worksite transmission risk (half, mean, or double), 500 stochastic simulations were run and the medians and interquartile ranges based on daily data are reported.

As a base case, the risk of transmission at construction worksites was assumed to be equal to the overall workplace transmission rate, estimated for all individuals aged 18 to 49 years. As a high-risk scenario, the transmission rate was double the base case. This might occur if construction work generally entails more frequent or extended physical contact or if interactions are elevated by workers migrating from nonessential worksites that are closed during stay-home orders to a smaller number of essential worksites. As a low-risk scenario, the workplace transmission was reduced by 50%, which might result from precautionary measures, including thorough cleaning of equipment between uses; wearing of protective equipment, such as gloves and masks; limits on the number of workers at a given worksite; and increased health surveillance on worksites, including daily temperature readings, rapid COVID-19 testing for workers with symptoms, contact tracing, and isolation of cases and known contacts of workers who test positive for COVID-19. The model assumed that Austin’s stay-home order reduced transmission overall by 73.3% until mid-August, when schools were scheduled to reopen. Model structure and a complete list of parameters are provided in eAppendix 2 in the Supplement.

In addition to running simulations, an analytic method was used to estimate the association of construction work with the *R_e_* of the virus in the surrounding community. As described in eAppendix 3 in the Supplement, an equation for the mean number of secondary infections from an infected construction worker was derived; it decomposes transmission events into those occurring at work and not at work. Based on the value of R_0_ estimated from hospitalization data during the Austin stay-home order, the level of transmission risk at construction worksites that would be expected to elevate the reproduction number to greater than 1 was determined.

### Estimating Relative Risk of Hospitalizations

To track risks in the construction workforce and other high-risk industries, Austin Public Health collects occupation information for patients hospitalized with COVID-19. Deidentified COVID-19 hospitalization line-list data were provided for this study by the 3 major hospital systems in the Austin metropolitan area (ie, Seton Healthcare Family; HCA Healthcare; and Baylor, Scott, and White Health). As of August 20, 2020, the 3 systems reported a total of 3536 COVID-19 hospitalizations, including 2267 patients between the ages of 18 and 64 years. This analysis was restricted to the 515 case reports (22.7%) in this age group that included occupation information. Of these, 77 (15.0%) reported working in construction.

Assuming a construction workforce of 50 000 in the Austin MSA and 1 380 000 total individuals between the ages of 18 and 64 years in Austin who are not construction workers, the confirmed cumulative hospitalization rate (*r_i_*) in each group can be estimated as *r_i_* = *h_i_* / *n*_i_ where *h_i_* corresponds to the number of hospitalized individuals in each group (ie, 77 for construction workers and 438 for the main population) and *n_i_* corresponds to their respective population sizes.

The relative risk (risk ratio; RR) of COVID-19 hospitalizations among construction workers is given by:^21^
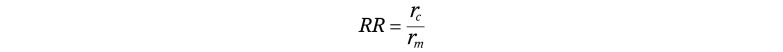
where *r_c_* and *r_m_* denote the cumulative COVID-19 hospitalization rates among construction workers and nonconstruction workers aged 18 to 64 years, respectively. Given the natural log of the RR is approximately normally distributed, the 95% CI for RR is given by:


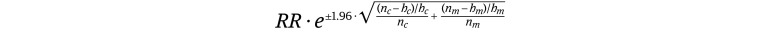


### Statistical Analysis

The relative risk of hospitalization of construction workers is computed using the methodology described earlier, following section 2 in the article by Katz et al.^21^ The mean and 95% confidence interval are reported. The analysis was conducted in Python version 3.7.6. Statistical significance was set at *P* < .05, and all tests were 2-tailed.

## Results

Fitting the COVID-19 transmission model to Austin area hospitalization data through May 3, 2020, suggests that when SARS-CoV-2 first emerged in Austin in February or March of 2020, the virus had a basic reproductive number of 4.14 (95% CI, 3.15-6.13) and a doubling time of 2.53 days. The March 24, 2020, stay-home order was associated with an estimated 73.3% (95% CI, 60.0%-80.0%) reduction in the local transmission rate to an *R_e_* of 0.96 (95% CI, 0.72-1.44) (Figure 1). Under these conditions, construction work would be expected to force the citywide reproduction number to greater than 1 if worksite transmission risk was just 14.2% higher than a typical workplace.

**Figure 2.**
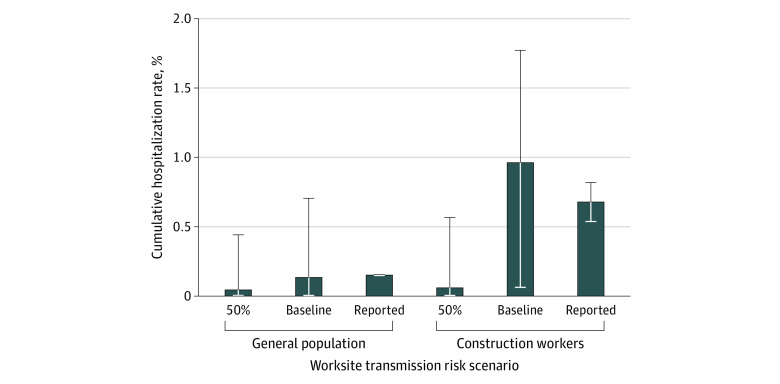
Projected Coronavirus Disease 2019 Hospitalizations in the Austin–Round Rock Metropolitan Area Projected and observed coronavirus disease 2019 hospitalization rates for the general population and construction workforce through August 20, 2020. Bars and error lines indicate 2.5th percentile, median, and 97.5th percentile across 500 stochastic simulations.

**Figure 3.**
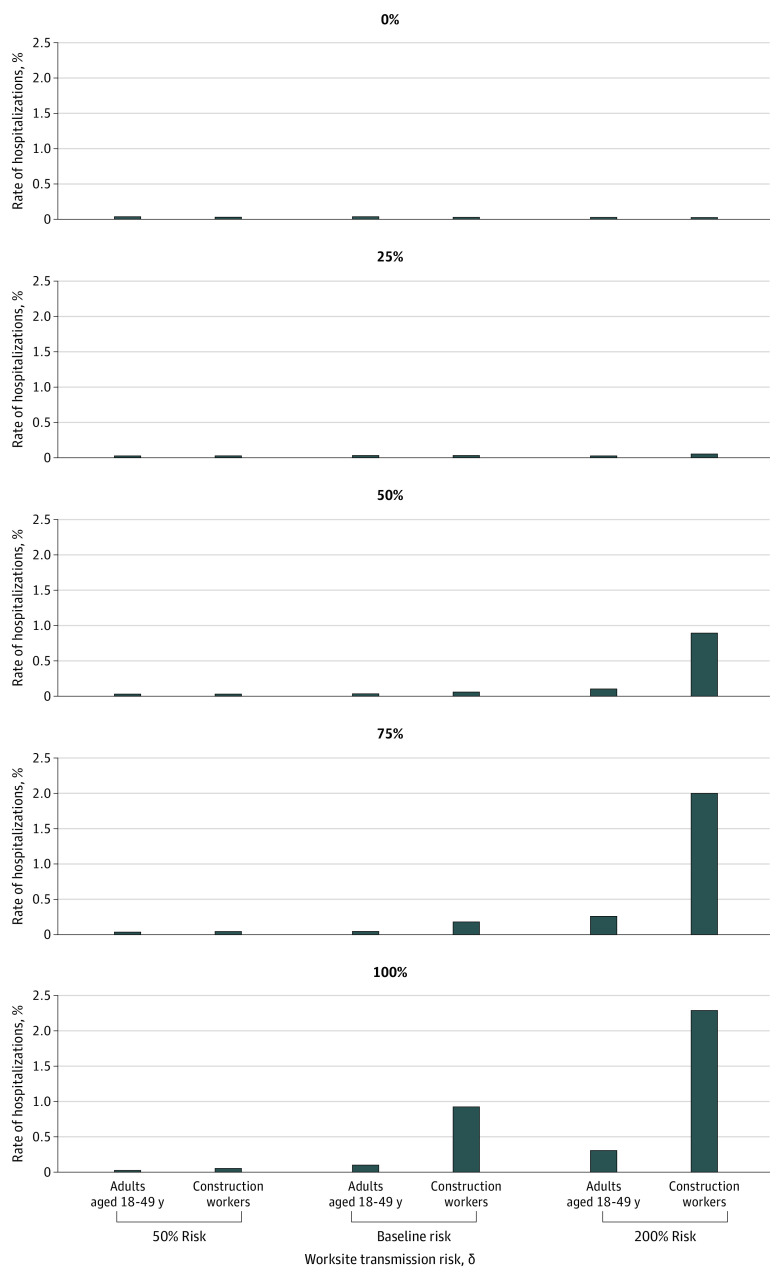
Projected Coronavirus Disease 2019 Hospitalization Rates in the Austin–Round Rock Metropolitan Statistical Area Under All Construction Work Scenarios Each graph represents a different proportion of the construction workforce being active. A bar for construction workers that is larger than the corresponding bar for the general population indicates a scenario where construction workers are at disproportionate risk for COVID-19 hospitalization. The relative risk of hospitalization in each scenario is given in eAppendix 4 in the Supplement.

**Figure 4.**
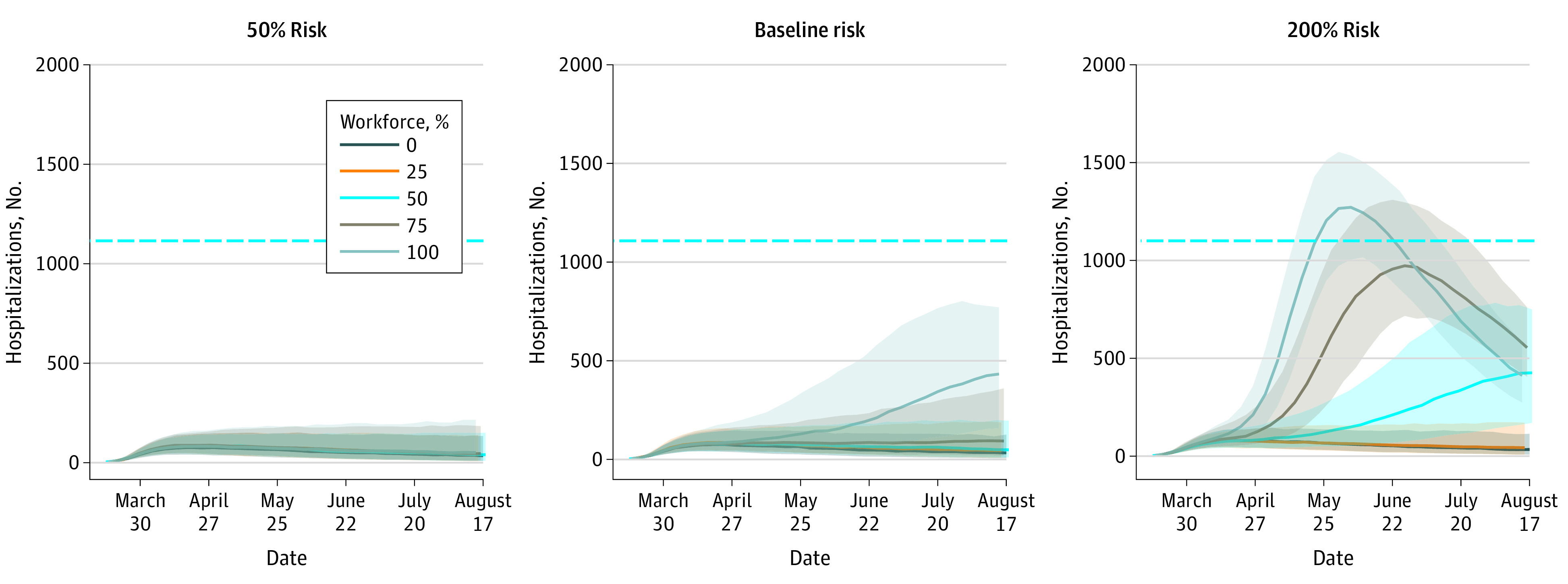
Projected Association of Construction Work With Coronavirus Disease 2019 Hospitalizations Through August 2020 Each graph represents a different proportion of the construction workforce being active. Shaded areas indicate the interquartile range across 500 stochastic simulations; horizontal blue line indicates the local hospital capacity (ie, 1100 beds). All parameters are provided in eAppendix 2 in the Supplement.

From the city of Austin’s COVID-19 hospital case reports through August 20, 2020, the median cumulative hospitalization rate was estimated at 1.5 per 1000 residents overall and 6.8 per 1000 construction workers (Figure 2). Based on model projections through the summer of 2020, it was estimated that allowing unrestricted construction work would be associated with an increase in the COVID-19 hospitalization rate from 0.38 per 1000 residents to 1.5 per 1000 residents overall. In the construction workforce, the risk would increase from 0.22 per 1000 construction workers to 9.3 per 1000 construction workers (Figure 3). Both the size of the workforce and the risk of infection at worksites were varied, and then the hospitalization risk among construction workers was estimated (Figure 3; eAppendix 4 in the Supplement). For each scenario, the RR of COVID-19 hospitalization was also calculated, ranging from 0.70 when no construction work was allowed to 8.65 when 100% of construction workers continued work with mean worksite transmission risk. Safety measures associated with at least a 50% decrease in worksite transmission were estimated to fully mitigate this increased risk. Similarly, reducing worksite transmission by at least 50% was associated with less transmission and fewer COVID-19 hospitalizations in the broader community. On the other hand, if worksite transmission risk was increased to 200% of its baseline level, construction work would be associated with an increase in hospitalizations (Figure 4). In general, reducing the number of workers and worksite risk through measures such as personal protective equipment, physical distancing, and generous sick leave act similarly, suggesting that strict worksite protocols could be used to counterbalance the risks of expanding the workforce.

As of mid-May, Austin Public Health had identified 19 clusters of at least 3 co-occurring confirmed COVID-19 cases in the construction industry, and by mid-July, 23 more had emerged.^22^ Based on COVID-19 hospital case reports through August 20, 2020, 77 of 515 hospitalized individuals (15.0%) aged 18 to 64 years with known occupation reported working in construction. Construction workers in Austin thus have an RR of COVID-19 hospitalization of 4.9 (95% CI, 3.8-6.2) compared with other occupational categories in the same age group, which is consistent with the projections made to support decision-making by the city of Austin in late March. Figure 3 suggests that this relative risk could correspond to multiple possible scenarios, including a small workforce (less than 50%) coupled with high worksite risk (ie, 200%) or large workforce (75%-100%) coupled with moderate worksite risk. Given that all construction was permitted following the Texas governor’s March 31, 2020, executive order,^10^ the latter scenario is a more plausible explanation for the observed data.

It was assumed that the proportion of construction workers with high-risk conditions was equal to that of the general population in the same age group. However, documented socioeconomic, occupational, and health susceptibilities among US construction workers suggest that the high-risk proportion may be elevated.^23,24^ A sensitivity analysis (eAppendix 4 in the Supplement) suggests that such disparities would amplify the disproportionate hospitalization risk among construction workers.^25^

## Discussion

In March 2020, as US cities and states rapidly enacted shelter-in-place orders to mitigate the emerging threat of COVID-19, local policy makers scrambled to determine which essential and semi-essential industries to exempt, without clear guidelines from state or federal authorities. Construction in Austin, Texas, was initially halted by a local order but then, 1 week later, deemed essential by a state order that allowed it to continue. Using a mathematical model to estimate the risks of maintaining key industries during the COVID-19 pandemic to both the industry workforce and surrounding metropolitan area, it appears that construction work in Austin during the spring 2020 lockdown was associated with a 5-fold greater COVID-19 hospitalization risk among workers and exacerbation of the local epidemic. However, stringent workplace safety measures could significantly mitigate these risks. These projections—which were originally made in response to a city request in March 2020—are borne out by an almost 5-fold higher COVID-19 hospitalization rate among construction workers relative to nonconstruction workers through mid-August (Figure 2).

This study demonstrates the feasibility of data-driven COVID-19 projections to inform local mitigation strategies and anticipate health care needs. It also provides evidence that opening industries that require daily contact between workers, especially indoors,^26^ can jeopardize the health of the workforce and community during waves of the COVID-19 pandemic. These findings prompted Austin to issue specific requirements for worksite management, including regular cleaning of shared equipment and common areas, management of the number of people on worksites, daily monitoring for symptoms, and record-keeping of individuals on every site for contact tracing.^9^ Cities throughout the United States have likewise specified guidelines, mandating COVID-19–specific training in multiple languages and worksite requirements beyond minimums from the Occupational Safety and Health Administration.^27^ Fully implementing and enforcing paid sick leave and protections from job loss related to COVID-19 could further improve containment.

The risk of infection on construction worksites is compounded by overlapping exposures in the construction workforce, which includes roughly 1 million undocumented workers across the United States.^28^ Nearly 30% of the construction workforce in the United States are Latinx individuals, 7% are Black individuals, and 5% belong to other minority groups; in Austin, 66% are Latinx individuals; 4%, Black individuals; and 3%, other minority groups.^29^ For these workers, occupational hazards are compounded by the increased risk of COVID-19 infection, hospitalization, and mortality among Black and Latinx individuals in the United States.^30^ Approximately 24% of all construction workers and nearly 48% of Latinx construction workers do not have health insurance^23^ and thus lack access to preventative care, have disproportionate comorbidities,^31^ and are less likely to seek timely and safe treatment for COVID-19 infections.^32^ Hospitalization risk may also be elevated by high rates of smoking and exposure to hazardous materials at worksites.^33^ Finally, transmission may be amplified by symptomatic cases continuing to work out of economic desperation and above-average sized households.^29,34^ The elevated risks and bleak projections hold broadly for low-paying industries with high-contact workplaces, such as the food processing plants and warehouses that have sustained devastating outbreaks in Texas, Indiana, and Delaware.^35,36,37^

Of note, these quantitative findings specifically pertain to COVID-19 risks associated with construction work in Austin, Texas, during the spring and summer of 2020. Extrapolating to other industries, communities, or periods requires a careful look at workforce size, workplace conditions, and the social network connecting the industry to the surrounding community. Nonetheless, the qualitative conclusions can be more broadly generalized. Opening industries with large locally integrated workforces that face higher than average workplace risk of infection can be risky. For any industry in any city, if an infected worker is likely to infect at least 1 coworker, then opening the industry will make containment impossible, unless the workforce is essentially cordoned off from the rest of the community. The excess disease burden among workers and their families and the spillover into the city may be substantial and exacerbated by overlapping risks in low-income communities. Industry-specific analyses, like those presented herein, can provide critical information for policy makers struggling to balance competing public health and economic needs during the COVID-19 pandemic. Moreover, they reflect the downstream benefits of providing resources and regulations to reduce exposure risks, including access to personal protective equipment, cohorting, and physical distancing at worksites as well as incentives for workers with underlying conditions or symptoms to stay home.

### Limitations

This study has limitations. The compartmental susceptible-exposed-infected-recovered model of COVID-19 transmission used to project construction-related risks makes several simplifying assumptions. The population is broken into 12 subgroups: 2 subgroups representing the construction workforce for 12 different age-specific and risk-specific groups. Although the transmission rates within and between subgroups vary widely, all individuals within a single subgroup are assumed to have identical infection rates. However, construction workers may face very different exposure risks depending on the type and size of their projects. More granular compartmental or agent-based models of COVID-19 transmission^38^ could provide sub–industry-level insights to inform staged reopenings and effectively targeted mitigation strategies.

In addition, the model assumes that the number of construction workers and the citywide contact patterns remain constant throughout the period of analysis. Although Texas has among the lowest seasonality in construction employment,^39^ extrapolating these findings to other cities or seasons should be done cautiously. The size of the construction workforce and worksite risk factors may depend on local pandemic mitigation strategies, which have varied considerably,^40^ and on seasonal conditions that impede outdoor construction, such as extreme heat, cold, or precipitation.^41^

Furthermore, the Austin hospitalization data includes occupation information for 515 of 2267 patients with COVID-19 aged 18 to 64 years. The reported estimates could be biased if construction workers were more or less likely to provide occupational data than other working adults. Data indicating whether hospitalized cases live in households with construction workers could provide further insight into the indirect associations of essential workforces with transmission rates in local communities.

Additionally, it was assumed that the risk of COVID-19 transmission at construction worksites was between half and twice that of the average worksite. For some types of construction and other industries, the risks could be outside of this range, depending on the number and nature of close contacts in the workplace and COVID-19 mitigation efforts.^42,43^

## Conclusions

The findings of this study suggest that the continuation of construction work in central Texas since the emergence of the pandemic was associated with increased transmission in the surrounding community and a 5-fold increased risk of hospitalization due to COVID-19 in a workforce with high proportions of workers who belong to racial and ethnic minority groups. As the United States navigates relaxing and enacting policies to mitigate the COVID-19 pandemic, communities should recognize the disproportionate burden of illness already experienced by workers in low-paying, high-contact industries. Temporarily closing semi-essential industries during pandemic waves, enhancing workplace safety policies, and providing paid sick leave could offset these risks and prevent further widening of disparities in COVID-19 morbidity and mortality.

## Notes

Supplement. **eAppendix 1. **Estimation of Age-Stratified Proportion of Population at High Risk of COVID-19 Complications **eAppendix 2. **Transmission Model **eAppendix 3. **Estimation of Secondary Infections at Work and Elsewhere **eAppendix 4. **Additional Simulation Results **eReferences.**

## References

[1] Troutman Pepper COVID-19 statewide and local executive orders and the construction industry. Published April 8, 2020. Accessed May 26, 2020. https://www.troutman.com/insights/covid-19-statewide-and-local-executive-orders-and-the-construction-industry.html

[2] ConstructConnect COVID-19 construction activity report. Updated April 30, 2020. Accessed May 26, 2020. https://www.constructconnect.com/covid-19-construction-activity-report

[3] NAHB Now DHS designates residential construction as “essential infrastructure business.” Published March 28, 2020 Accessed May 26, 2020. http://nahbnow.com/2020/03/dhs-designates-residential-construction-as-essential-infrastructure-business/

[4] US Bureau of Labor Statistics May 2019 national occupational employment and wage estimates. Updated March 31, 2020. Accessed May 7, 2020. https://www.bls.gov/oes/current/oes_nat.htm

[5] O*Net Online Details report for: 47-2061.00—construction laborers. Accessed June 6, 2020. https://www.onetonline.org/link/details/47-2061.00

[6] US Bureau of Labor Statistics Labor force statistics from the current population survey. Updated January 22, 2020 Accessed June 20, 2020. https://www.bls.gov/cps/cpsaat18.htm

[7] Velasco-Mondragon E, Jimenez A, Palladino-Davis AG, Davis D, Escamilla-Cejudo JA Hispanic health in the USA: a scoping review of the literature. Public Health Rev. 2016;37:31. doi:10.1186/s40985-016-0043-2 29450072 PMC5809877

[8] Stokes EK, Zambrano LD, Anderson KN, Coronavirus disease 2019 case surveillance—United States, January 22-May 30, 2020. MMWR Morb Mortal Wkly Rep. 2020;69(24):759-765. doi:10.15585/mmwr.mm6924e2 32555134 PMC7302472

[9] Mayor of the City of Austin Stay Home—Work Safe: Order 2020200324-007. Published March 24, 2020. Accessed October 2, 2020. http://www.austintexas.gov/sites/default/files/files/Order%2020200324-007%20-%20Stay%20Home%20-%20Work%20Safe.pdf

[10] Mayor of the City of Austin Supplemental guidance based on executive order No.GA-14. Published April 2, 202. Accessed October 2, 2020. http://austintexas.gov/sites/default/files/files/GUIDANCE%20-%20Response%20to%20GA-14%20%20FINAL.pdf

[11] Pasco R, Du Z, Wang X, Petty M, Fox SJ, Meyers LA. COVID-19 in Austin, Texas: epidemiological assessment of construction work. Accessed May 8, 2020. https://cid.utexas.edu/sites/default/files/cid/files/covid-19_austin_construction_workforce-meyers_ut-040520.pdf?m=1586183590

[12] US Census Bureau American Community Survey (ACS). Accessed November 19, 2019. https://www.census.gov/programs-surveys/acs

[13] US Centers for Disease Control and Prevention 500 Cities Project: local data for better health. Published December 5, 2019 Accessed March 19, 2020. https://www.cdc.gov/500cities/index.htm

[14] US Centers for Disease Control and Prevention HIV surveillance reports. Accessed October 2, 2020. https://www.cdc.gov/hiv/library/reports/hiv-surveillance.html

[15] Morgan OW, Bramley A, Fowlkes A, Morbid obesity as a risk factor for hospitalization and death due to 2009 pandemic influenza A(H1N1) disease. PLoS One. 2010;5(3):e9694. doi:10.1371/journal.pone.0009694 20300571 PMC2837749

[16] Sturm R, Hattori A Morbid obesity rates continue to rise rapidly in the United States. Int J Obes (Lond). 2013;37(6):889-891. doi:10.1038/ijo.2012.159 22986681 PMC3527647

[17] Martin JA, Hamilton BE, Osterman MJK, Driscoll AK, Drake P Births: final data for 2017. Natl Vital Stat Rep. 2018;67(8):1-50. 30707672

[18] Jatlaoui TC, Boutot ME, Mandel MG, Abortion surveillance—United States, 2015. MMWR Surveill Summ. 2018;67(13):1-45. doi:10.15585/mmwr.ss6713a1 30462632 PMC6289084

[19] Austin Chamber of Commerce Workforce overview. Accessed March 30, 2020. https://www.austinchamber.com/economic-development/austin-profile/workforce/overview

[20] Diekmann O, Heesterbeek JAP, Roberts MG The construction of next-generation matrices for compartmental epidemic models. J R Soc Interface. 2010;7(47):873-885. doi:10.1098/rsif.2009.0386 19892718 PMC2871801

[21] Katz D, Baptista J, Azen SP, Pike MC Obtaining confidence intervals for the risk ratio in cohort studies. Biometrics. 1978;34(3):469-474. doi:10.2307/2530610

[22] Wilson MD, Korte L For Austin construction crews, coronavirus choice can be lives vs livelihoods. Updated August 3, 2020. Accessed September 7, 2020. https://www.statesman.com/news/20200724/for-austin-construction-crews-coronavirus-choice-can-be-lives-vs-livelihoods

[23] Brown S, Brooks RD, Dong XS Health insurance coverage in the construction industry. Published April 2020. Accessed October 2, 2020. https://www.cpwr.com/sites/default/files/publications/DataBulletin-April-2020.pdf

[24] The Center for Construction Research and Training Health risk factors and chronic illnesses among construction workers. Accessed October 2, 2020. https://www.cpwr.com/wp-content/uploads/publications/CB-page-54.pdf

[25] Engel J. Austin-Travis County reveals new COVID-19 clusters while hospitalizations remain flat. KXAN Austin. Published online May 19, 2020. Accessed June 20, 2020. https://www.kxan.com/news/coronavirus/austin-travis-county-reveals-new-covid-19-cluster-data-as-economy-reopens/

[26] Leclerc QJ, Fuller NM, Knight LE, Funk S, Knight GM; CMMID COVID-19 Working Group What settings have been linked to SARS-CoV-2 transmission clusters? Wellcome Open Res. 2020;5:83. doi:10.12688/wellcomeopenres.15889.2 32656368 PMC7327724

[27] Occupational Safety and Health Administration Guidance on preparing workplaces for COVID-19. Accessed October 2, 2020. https://www.osha.gov/Publications/OSHA3990.pdf

[28] Workers Defense Project Build a better nation: a case for comprehensive immigration reform. Accessed October 2, 2020. https://www.workersdefense.org/IMMIGRATION%20wdp%20color%20FINAL.pdf

[29] US Census Bureau Public Use Microdata Sample (PUMS). Accessed May 27, 2020. https://www.census.gov/programs-surveys/acs/data/pums.html

[30] US Centers for Disease Control and Prevention People with certain medical conditions. Published May 12, 2020 Accessed May 14, 2020. https://www.cdc.gov/coronavirus/2019-ncov/need-extra-precautions/groups-at-higher-risk.html

[31] The Center for Construction Research and Training Chart book (6th edition): health indicators and services—health risk factors and chronic illnesses among construction workers. Accessed May 14, 2020. https://www.cpwr.com/chart-book-6th-edition-health-indicators-and-services-health-risk-factors-and-chronic-illnesses

[32] Page KR, Venkataramani M, Beyrer C, Polk S Undocumented U.S. Immigrants and COVID-19. N Engl J Med. 2020;382(21):e62. doi:10.1056/NEJMp2005953 32220207

[33] Wang X, Dong XS, Welch L, Largay J Respiratory cancer and non-malignant respiratory disease-related mortality among older construction workers—findings from the health and retirement study. Occup Med Health Aff. 2016;4:235. doi:10.4172/2329-6879.1000235 27500180 PMC4975376

[34] Narea N For immigrants without legal status, federal coronavirus relief is out of reach. Vox. Published May 5, 2020 Accessed May 26, 2020. https://www.vox.com/2020/5/5/21244630/undocumented-immigrants-coronavirus-relief-cares-act

[35] Wired. Why meatpacking plants have become COVID-19 hot spots. Published online May 7, 2020. Accessed May 7, 2020. https://www.wired.com/story/why-meatpacking-plants-have-become-covid-19-hot-spots/

[36] Legan M 1,000+ Tyson infections and one hospital: Cass Co. braces for even more COVID Cases. Indiana Public Media. Published May 1, 2020. Accessed May 7, 2020. https://indianapublicmedia.org/news/1,000+-cases-and-one-hospital-cass-county-braces-for-even-more-covid-cases.php

[37] Sternberg S Chicken plants take center stage in Delaware’s coronavirus fight. US News & World Report. Published May 5, 2020 Accessed May 27, 2020. https://www.usnews.com/news/healthiest-communities/articles/2020-05-05/chicken-plants-take-center-stage-in-delaware-coronavirus-fight

[38] Lee S, Zabinsky ZB, Kofsky SM, Liu S COVID-19 pandemic response simulation: impact of non-pharmaceutical interventions on ending lockdowns. medRxiv. Preprint posted May 4, 2020. doi:10.1101/2020.04.28.20080838

[39] Geremew F, Gourio F Seasonal and business cycles of US employment. Federal Reserve Bank of Chicago. Accessed August 25, 2020. https://www.chicagofed.org/publications/economic-perspectives/2018/3

[40] Lo Bue LB, Stockwell MD, Argyris AM, Dye EJ Government orders and guidelines. Updated July 27, 2020. Accessed October 2, 2020. https://www.pillsburylaw.com/images/content/1/3/130443/COVID-19-Construction-Chart.pdf

[41] Ibbs W, Vaughan C Change and the loss of productivity in construction: a field guide. Ibbs Consulting Group. Published January 27, 2012. Accessed October 2, 2020. http://www.theibbsconsultinggroup.com/uploads/Changes_Field_Guide_July_2013.pdf

[42] Nishiura H, Oshitani H, Kobayashi T, Closed environments facilitate secondary transmission of coronavirus disease 2019 (COVID-19). medRxiv. Preprint posted March 3, 2020. doi:10.1101/2020.02.28.20029272

[43] O*Net Online Work context: exposed to disease or infections. Accessed September 10, 2020. https://www.onetonline.org/find/descriptor/result/4.C.2.c.1.b

