# Supplementary material for: Estimated Association of Construction Work With Risks of COVID-19 Infection and Hospitalization in Texas
Source: JAMA Netw Open. 2020 Oct 29;3(10):e2026373. doi: 10.1001/jamanetworkopen.2020.26373 (PMC7596583; doi:10.1001/jamanetworkopen.2020.26373)
Supplement: Supplement. — eAppendix 1. Estimation of Age-Stratified Proportion of Population at High Risk of COVID-19 Complications eAppendix 2. Transmission Model eAppendix 3. Estimation of Secondary Infections at Work and Elsewhere eAppendix 4. Additional Simulation Results eReferences. [file jamanetwopen-e2026373-s001.pdf]

Supplemental Online Content

Pasco RF, Fox SJ, Johnston SC, Pignone M, Meyers LA. Estimated association of construction work with risks of COVID-19 infection and hospitalization in Texas. *JAMA Netw Open*. 2020;3(10):e2026373. doi:10.1001/jamanetworkopen.2020.26373

eAppendix 1. Estimation of Age-Stratified Proportion of Population at High Risk of COVID-19 Complications
eAppendix 2. Transmission Model
eAppendix 3. Estimation of Secondary Infections at Work and Elsewhere
eAppendix 4. Additional Simulation Results
eReferences.

This supplemental material has been provided by the authors to give readers additional information about their work.

© 2020 Pasco RF et al. *JAMA Network Open.*

eAppendix 1. Estimation of Age-Stratified Proportion of Population at High Risk of COVID-19 Complications

We estimate age-specific proportions of the population at high risk of complications from COVID-19 based on data for Austin, TX and Round-Rock, TX from the CDC's 500 cities project (Figure S1).[5] We assume that high risk conditions for COVID-19 are the same as those specified for influenza by the CDC.[20] The CDC's 500 cities project provides city-specific estimates of prevalence for several of these conditions among adults.[26] The estimates were obtained from the 2015-2016 Behavioral Risk Factor Surveillance System (BRFSS) data using a small-area estimation methodology called multi-level regression and poststratification.[21,22] It links geocoded health surveys to high spatial resolution population demographic and socioeconomic data.[22]

Estimating high-risk proportions for adults. To estimate the proportion of adults at high risk for complications, we use the CDC's 500 cities data, as well as data on the prevalence of HIV/AIDS, obesity and pregnancy among adults (Table S1).

The CDC 500 cities dataset includes the prevalence of each condition on its own, rather than the prevalence of multiple conditions (e.g., dyads or triads). Thus, we use separate co-morbidity estimates to determine overlap. Reference[27] about chronic conditions gives US estimates for the proportion of the adult population with 0, 1 or 2+ chronic conditions, per age group. Using this and the 500 cities data we can estimate the proportion of the population p_{HR} in each age group in each city with at least one chronic condition listed in the CDC 500 cities data (Table S2) putting them at high-risk for influenza complications.

HIV: We use the data from table 20a in CDC HIV surveillance report[6] to estimate the population in each risk group living with HIV in the US (last column, 2015 data). Assuming independence between HIV and other chronic conditions, we increase the proportion of the population at high-risk for influenza to account for individuals with HIV but no other underlying conditions.

Morbid obesity: A BMI over 40kg/m^2 indicates morbid obesity and is considered high risk for influenza. The 500 Cities Project reports the prevalence of obese people in each city with BMI over 30kg/m^2 (not necessarily morbid obesity). We use the data from table 1 in Sturm and Hattori[8] to estimate the proportion of people with BMI>30 that actually have BMI>40 (across the US); we then apply this to the 500 Cities obesity data to estimate the proportion of people who are morbidly obese in each city. Table 1 of Morgan et al.[7] suggests that 51.2% of morbidly obese adults have at least one other high risk chronic condition, and update our high-risk population estimates accordingly to account for overlap.

Pregnancy: We separately estimate the number of pregnant women in each age group and each city, following the methodology in CDC reproductive health report.[9] We assume independence between any of the high-risk factors and pregnancy, and further assume that half the population are women.

Estimating high-risk proportions for children. Since the 500 Cities Project only reports data for adults 18 years and older, we take a different approach to estimating the proportion of children at high risk for severe influenza. The two most prevalent risk factors for children are asthma and obesity; we also account for childhood diabetes, HIV and cancer.

From Miller et al.,[28] we obtain national estimates of chronic conditions in children. For asthma, we assume that variation among cities will be similar for children and adults. Thus, we use the relative prevalences of asthma in adults to scale our estimates for children in each city. The prevalence of HIV and cancer in children are taken from CDC HIV surveillance report[6] and cancer research report,[29] respectively.

© 2020 Pasco RF et al. *JAMA Network Open.*

We first estimate the proportion of children having either asthma, diabetes, cancer or HIV (assuming no overlap in these conditions). We estimate city-level morbid obesity in children using the estimated morbid obesity in adults multiplied by a national constant ratio for each age group estimated from Hales et al.,[30] this ratio represents the prevalence in morbid obesity in children given the one observed in adults. From Morgan et al.,[7] we estimate that 25% of morbidly obese children have another high-risk condition and adjust our final estimates accordingly.

Resulting estimates. We compare our estimates for the Austin-Round Rock Metropolitan Area to published national-level estimates[31] of the proportion of each age group with underlying high risk conditions (Table S2). The biggest difference is observed in older adults, with Austin having a lower proportion at risk for complications for COVID-19 than the national average; for 25-39 year olds the high risk proportion is slightly higher than the national average.

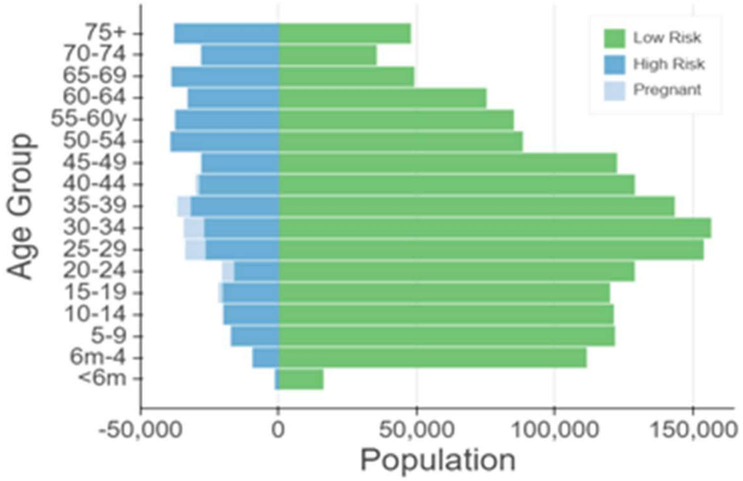

Figure S1. Demographic and risk composition of the Austin-Round Rock MSA. Bars indicate age-specific population sizes, separated by low risk, high risk, and pregnant. High risk is defined as individuals with cancer, chronic kidney disease, COPD, heart disease, stroke, asthma, diabetes, HIV/AIDS, and morbid obesity, as estimated from the CDC 500 Cities Project,[5] reported HIV prevalence,[6] and reported morbid obesity prevalence,[7,8] corrected for multiple conditions. The population of pregnant women is derived using the CDC's method combining fertility, abortion and fetal loss rates.[32–34]

Table S1. High-risk conditions for influenza and data sources for prevalence estimation

**Condition**	**Data source**
Cancer (except skin), chronic kidney disease, COPD, coronary heart disease, stroke, asthma, diabetes	CDC 500 cities[5]
HIV/AIDS	CDC HIV Surveillance report[6]
Obesity	CDC 500 cities,[5] Sturm and Hattori[8] Morgan et al.[7]
Pregnancy	National Vital Statistics Reports[32] and abortion data[33]

Table S2. Comparison between published national estimates and Austin-Round Rock MSA estimates of the percent of the population at high-risk of COVID-19 complications

Age Group	National estimates[30]	Austin (excluding pregnancy)	Pregnant women (proportion of age group)
0 to 6 months	NA	6.8	-
6 months to 4 years	6.8	7.4	-
5 to 9 years	11.7	11.6	-
10 to 14 years	11.7	13.0	-
15 to 19 years	11.8	13.3	1.7
20 to 24 years	12.4	10.3	5.1
25 to 34 years	15.7	13.5	7.8
35 to 39 years	15.7	17.0	5.1
40 to 44 years	15.7	17.4	1.2
45 to 49 years	15.7	17.7	-
50 to 54 years	30.6	29.6	-
55 to 60 years	30.6	29.5	-
60 to 64 years	30.6	29.3	-
65 to 69 years	47.0	42.2	-
70 to 74 years	47.0	42.2	-
75 years and older	47.0	42.2	-

Correlation with other risk factors for severe COVID-19 disease. Several recent studies have identified risk factors for severe outcomes from COVID-19 infection. While there is substantial overlap with those included in the model, there are a few that are not explicitly considered. For instance, Killerby et al.[35] and Hamer et al.[36] suggest that black race, lack of insurance, male sex, smoking, obesity, and physical inactivity increase risk.

In order to assess the adequacy of our model, we measured the correlation between the prevalence of high risk conditions assumed by our model and the prevalence of other COVID-19 high risk conditions, namely obesity, physical activity, insurance rates, and smoking rates across the cities included in the CDC 500 Cities's dataset.[5] We find a high degree of overlap, with uninsured rates having the lowest correlation (Figure S2).

© 2020 Pasco RF et al. *JAMA Network Open.*

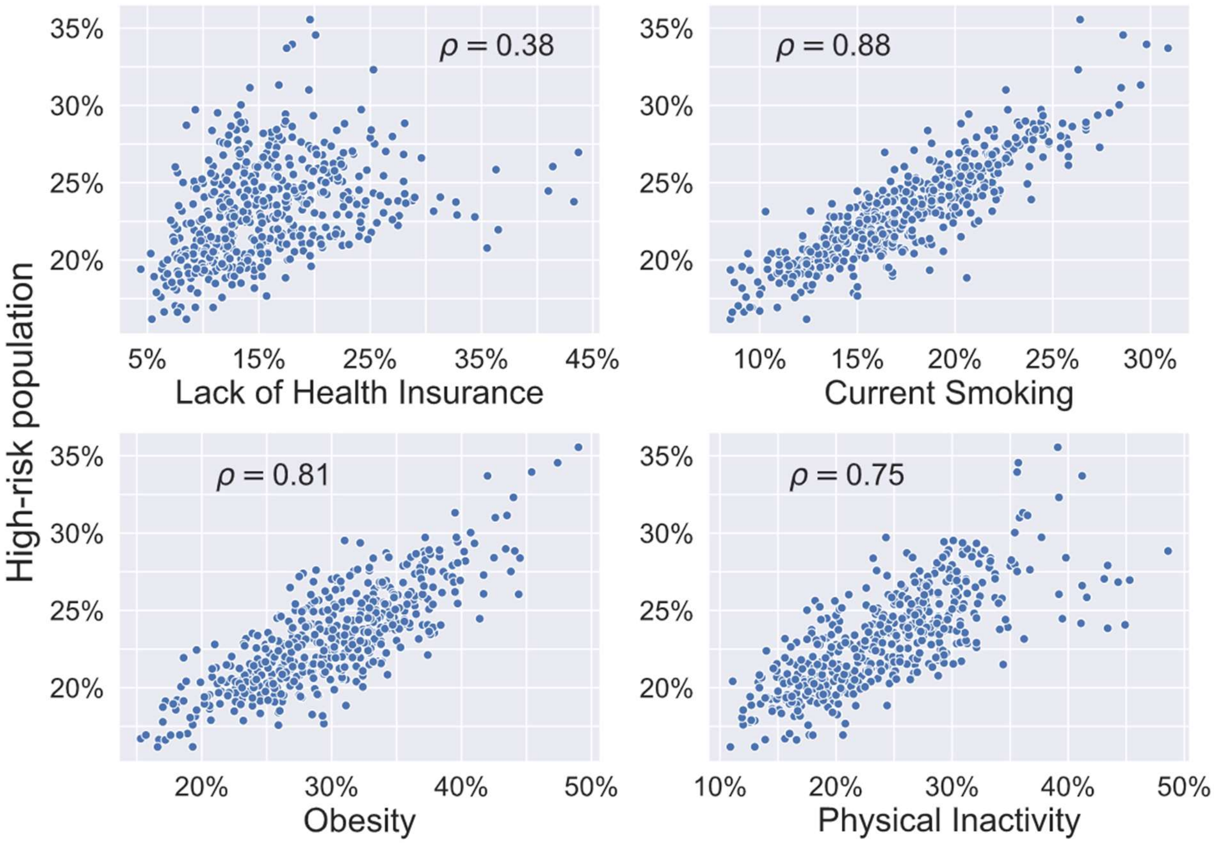

Figure S2. Correlation between high risk proportions assumed in the model (y-axis) and other COVID-19 high-risk factors (x-axes): lack of health insurance, smoking, obesity, and physical inactivity prevalence. Each point corresponds to a city included in the CDC's 500 cities project. Numbers in plots are the Pearson correlation coefficients.

© 2020 Pasco RF et al. *JAMA Network Open.*

eAppendix 2. Transmission Model

2.1. Structure

The model structure is diagrammed in Figure S3 and described in the equations below.
For each age and risk group, we build a separate set of compartments to model the transitions between the states: susceptible (S), exposed (E), pre-symptomatic infectious (P^Y), pre-asymptomatic infectious (P^A), symptomatic infectious (I^Y), asymptomatic infectious (I^A), symptomatic infectious that are hospitalized (I^H), recovered (R), and deceased (D). The symbols S, E, P^Y, P^A ,I^Y, I^A, I^H, R, and D denote the number of people in that state in the given age/risk group and the total size of the age/risk group is $N = S + E + P^Y + P^A + I^Y + I^A + I^H + R + D$.
The model for individuals in age group a and risk group r is given by:

$$\frac{dS_{a,r}}{dt} = -S_{a,r} \cdot \sum_{i \in A} \sum_{j \in K} (I_{i,j}^Y \omega^Y + I_{i,j}^A \omega^A + P_{i,j}^Y \omega^{PY} + P_{i,j}^A \omega^{PA}) \beta \phi_{a,i} / N_i$$

$$\frac{dE_{a,r}}{dt} = S_{a,r} \cdot \sum_{i \in A} \sum_{j \in K} (I_{i,j}^Y \omega^Y + I_{i,j}^A \omega^A + P_{i,j}^Y \omega^{PY} + P_{i,j}^A \omega^{PA}) \beta \phi_{a,i} / N_i - \sigma E_{a,r}$$

$$\frac{dP_{a,r}^A}{dt} = (1 - \tau) \sigma E_{a,r} - \rho^A P_{a,r}^A$$

$$\frac{dP_{a,r}^Y}{dt} = \tau \sigma E_{a,r} - \rho^Y P_{a,r}^Y$$

$$\frac{dI_{a,r}^A}{dt} = \rho^A P_{a,r}^A - \gamma^A I_{a,r}^A$$

$$\frac{dI_{a,r}^Y}{dt} = \rho^Y P_{a,r}^Y - (1 - \pi) \gamma^Y I_{a,r}^Y - \pi \eta I_{a,r}^Y$$

$$\frac{dI_{a,r}^H}{dt} = \pi \eta I_{a,r}^Y - (1 - \nu) \gamma^H I_{a,r}^H - \nu \mu I_{a,r}^H$$

$$\frac{dR_{a,r}}{dt} = \gamma^A I_{a,r}^A + (1 - \pi) \gamma^Y I_{a,r}^Y + (1 - \nu) \gamma^H I_{a,r}^H$$

$$\frac{dD_{a,r}}{dt} = \nu \mu I_{a,r}^H$$

where A and K are all possible age and risk groups, $\omega^A, \omega^Y, \omega^{PA}, \omega^{PY}$ are the relative infectiousness of the I^A, I^Y, I^{PA}, I^{PY} compartments, respectively, β is transmission rate, $\phi_{a,i}$ is the mixing rate between age group $a, i \in A$, $\gamma^A, \gamma^Y, \gamma^H$ are the recovery rates for the I^A, I^Y, I^H compartments, respectively, σ is the exposed rate, ρ^A, ρ^Y are the pre-(a)symptomatic rates, τ is the symptomatic ratio, π is the proportion of symptomatic individuals requiring hospitalization, η is rate at which hospitalized cases enter the hospital following symptom onset, ν is mortality rate for hospitalized cases, and μ is rate at which terminal patients die.
We model stochastic transitions between compartments using the τ-leap method[2,3] with key parameters given in Table S3. Assuming that the events at each time-step are independent and do not impact the underlying transition rates, the numbers of each type of event should follow Poisson distributions with means equal to the rate parameters. We thus simulate the model according to the following equations:

$$S_{a,r}(t+1) - S_{a,r}(t) = -P_1$$

$$E_{a,r}(t+1) - E_{a,r}(t) = P_1 - P_2$$

$$P_{a,r}^A(t+1) - P_{a,r}^A(t) = (1 - \tau) P_2 - P_3$$

$$P_{a,r}^Y(t+1) - P_{a,r}^Y(t) = \tau P_2 - P_4$$

$$I_{a,r}^A(t+1) - I_{a,r}^A(t) = P_3 - P_5$$

$$I_{a,r}^Y(t+1) - I_{a,r}^Y(t) = P_4 - P_6 - P_7$$

© 2020 Pasco RF et al. *JAMA Network Open.*

$$I^H_{a,r}(t+1) - I^H_{a,r}(t) = P_7 - P_8 - P_9$$
$$R_{a,r}(t+1) - R_{a,r}(t) = P_5 + P_6 + P_8$$
with
$$P_1 \sim \text{Pois}(S_{a,r}(t)F_{a,r}(t))$$
$$P_2 \sim \text{Pois}(\sigma E_{a,r}(t))$$
$$P_3 \sim \text{Pois}(\rho^A P^A_{a,r}(t))$$
$$P_4 \sim \text{Pois}(\rho^Y P^Y_{a,r}(t))$$
$$P_5 \sim \text{Pois}(\gamma^A I^A_{a,r}(t))$$
$$P_6 \sim \text{Pois}((1-\pi)\gamma^Y I^Y_{a,r}(t))$$
$$P_7 \sim \text{Pois}(\pi\eta I^Y_{a,r}(t))$$
$$P_8 \sim \text{Pois}((1-\nu)\gamma^H I^H_a)$$
$$P_9 \sim \text{Pois}(\nu\mu I^H_{a,r}(t))$$

and where $F_{a,r}$ denotes the force of infection for individuals in age group a and risk group r and is given by:

$$F_{a,r}(t) = \sum_{i\in A}\sum_{j\in K}(I^Y_{i,r}(t)\omega^Y + I^A_{i,r}(t)\omega^A + P^Y_{i,j}(t)\omega^{PY} + P^A_{i,j}(t)\omega^{PA})\beta_{a,i}\phi_{a,i}/N_i$$

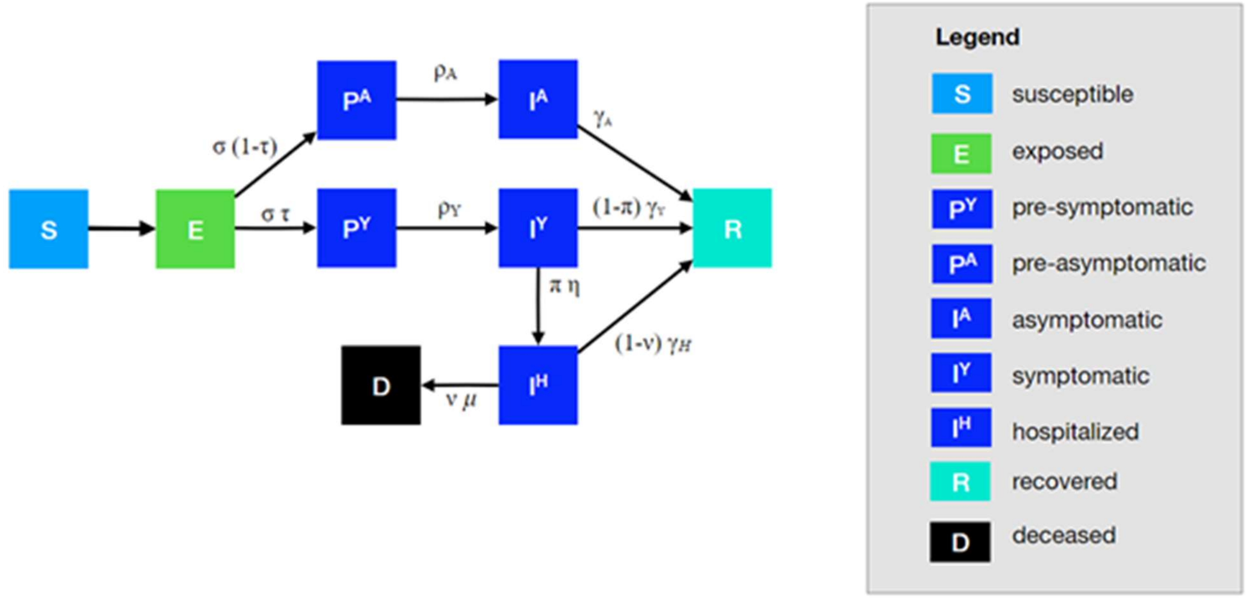

Figure S3. Compartmental model of COVID-19 transmission
Each subgroup (defined by age and risk) is modeled with a separate set of compartments. Upon infection, susceptible individuals (S) progress to exposed (E) and then to either pre-symptomatic infectious (P^Y) or pre-asymptomatic infectious (P^A) from which they move to symptomatic infectious (I^Y) and asymptomatic infectious (I^A) respectively. All asymptomatic cases eventually progress to a recovered class where they remain protected from future infection (R); symptomatic cases are either hospitalized (I^H) or recover. Mortality (D) varies by age group and risk group and is assumed to be preceded by hospitalization.

© 2020 Pasco RF et al. *JAMA Network Open.*

2.2. Parameters

Individuals were initially separated into five age groups: 0-4, 5-7, 18-49, 50-64, and 65+ years old based on population data for the five-county Austin-Round Rock Metropolitan Area from the 2017 American Community Survey.[4] Each age group was divided into a low-risk and high-risk group, based on the prevalence of chronic conditions estimated for the Austin population (Fig. S2).[5–8] We also estimated the proportion of pregnant women in each age group as a special risk class.[9] All individuals were assumed to be susceptible to the disease.

There are an estimated 50,000 construction workers in the Austin metropolitan area representing over 4% of the labor force,[10] not accounting for undocumented workers. We assumed all construction workers to be in the 18-49 group for simplicity, with the same low/high-risk distribution, and we modeled them as a 6th separate group with specific contacts, adding an extra row and an extra column to the contact matrices specifically for construction workers so as to be able to model their specific contact patterns as intended. All details on the construction of the adjusted contact matrices as well as the derived matrices are provided in sections 2.3-2.5. In order to keep the total population constant, we reduced the number of individuals in the 18-49 group according to the number of construction workers.

Infected individuals were modeled to enter an incubation period where they were symptom-free and not yet infectious. They then enter a pre-(a)symptomatic compartment where they do not present symptoms yet but they already transmit the disease,[11] with distinction between asymptomatic and symptomatic starting in this compartment. Individuals then progressed accordingly to either a symptomatic or asymptomatic compartment. Asymptomatic individuals were assumed to have the same pre-symptomatic and infectious period as symptomatic individuals but lower infectiousness.[12] The rates at which symptomatic cases were moved to a hospitalized compartment and died depended on both age and risk group. Recovered individuals were considered fully immune.

All model parameters are provided in Tables S3-S5 and were based on published estimates from COVID-19 studies as well as input from the US CDC and City of Austin. Age-specific contact rates were estimated using contact matrices published by Prem et al. and are adjusted to model school closures and various levels of social distancing.[13] The pre-symptomatic period was sampled from a triangular distribution from 1.9 days to 3.9 days with mean of 2.9 days[11,14] and the infectious period was sampled from a triangular distribution from 3 days to 5 days with mean of 4 days,[11] with a mean generation interval estimated at 5.8 days.[14] We assumed the asymptomatic ratio to be 43%[15] with 44% of infections arising from pre-symptomatic transmission during the incubation period.[11] Following the CDC's planning scenarios and observed data from the 2009 H1N1 pandemic in the United States, we assumed that the infection hospitalization rate and infection fatality rate was ten times higher in high-risk than low-risk individuals, within each age group. The average length of stay of 11 days in the hospital for people who survive and recover and 7.8 days for people who die is estimated using arrival and departure data from the Seton system in Austin.[16,17]

We estimate β and d simultaneously using a nonlinear least squares fitting procedure in the SciPy/Python package[18] For a given pair of β and d, we run a deterministic simulation of our model assuming central values for each parameter. Using a trust region method, the algorithm finds values of β and d that minimize the sum of squared daily differences between the simulated ($\hat{H}_t$) and actual (H_t) daily hospitalizations from March 13, 2020 through May 3, 2020: $S(\beta, d) = \sum_t (H_t - \hat{H}_t)^2$.

We calculated 95% confidence intervals for the social distancing parameter d indirectly by running 500 stochastic simulations for each of the following possible values of d': 0.0, 0.05, ..., 0.95, 1.0. For each value of d', we conducted the following analysis to determine if d' lies inside the 95% confidence interval for d.

© 2020 Pasco RF et al. *JAMA Network Open.*

- For all simulations, we calculate the day-to-day difference in hospitalizations (i.e., heads in beds) during the period following the *Stay Home-Work Safe* order: $\hat{z}_t = \hat{H}_t - \hat{H}_{t-1}$. We do the same for the actual data: $z_t = H_t - H_{t-1}$.

- We compute the 95% prediction interval for $\hat{z}_t$ across all 500 stochastic simulations for d' for each day *t*.

- We then conduct a test of the null hypothesis $H_0 : d' = d$. Under this null hypothesis, we would expect roughly 95% of the observed data (z_t) to fall within the 95% prediction band for $\hat{z}_t$ that we constructed from our simulations. By analyzing the day-to-day difference in hospitalizations rather than daily hospitalizations, we can assume that the data are independent from one day to the next. Then the expected number of observed values contained in the 95% prediction band is given by the binomial expression:
$$N_{\text{contained}} \sim B\left(N_{\text{points}}, 0.95\right)$$
where $N_{\text{contained}}$ is the number of data points contained within the 95% prediction band and N_{points} is the total number of data points (i.e., days).
- If the binomial probability of $N_{\text{contained}}$ is less than 0.05, we reject the null hypothesis $H_0 : d' = d$.

To construct a 95% confidence interval for d we take the minimum and maximum d' for which we did not reject the null hypothesis $H_0 : d' = d$.

In order to obtain 95% confidence intervals for the basic reproductive number R_0 and the doubling time we calculated the 95% confidence interval for the transmission rate β using the same methodology as that used for the social distancing parameter d. We fixed the social distancing parameter at its best estimated value and ran 500 stochastic simulations for each of the possible values of β': $0.2 \cdot \hat{\beta}$, $0.22 \cdot \hat{\beta}$, ... , $2 \cdot \hat{\beta}$ with $\hat{\beta} = 0.053$ our best estimate for the transmission rate. We then conducted the same analysis as that described above and the 95% confidence interval for β is obtained by taking the minimum and maximum β' for which we did not reject the null hypothesis $H_0 : \beta' = \beta$.

The confidence intervals for R_0 and the doubling time are then obtained by using the confidence interval bounds of β as input to the system's Next Generation Matrix.

Table S3. Initial conditions, school closures and social distancing policies

**Variable**	**Settings**
Initial day of simulation	2/29/2020 (Best fit[a])
Initial infection number	5 pre-symptomatic cases in 18-49y age group
Trigger to close school	3/19/2020
Closure Duration	Until start of 2020-2021 school year (8/17/20)

© 2020 Pasco RF et al. *JAMA Network Open.*

p_w: proportion construction workers who continue to work	Five scenarios: [0, 0.25, 0.5, 0.75, 1]
δ: contact rates at work between active construction workers are equal to baseline contact rates for 18-49 year old at work multiplied by a scaling factor	Three scenarios for scaling factor: [0.5, 1, 2]
d: Reduction of contacts after *Stay Home* order	0.733 (Fit[b] to Austin's observed hospitalizations up to 5/3/2020)
work_CW: contact matrix of construction workers	Contact matrices with construction workers provided in Tables S10-S13<br>work_CW = work - work(1:5, 1:5)
Age-specific and day-specific contact rates	Original home, work, other and school matrices provided in Tables S6-S9<br><br>Normal weekday = home + work + other + school + p_w*δ*work_CW<br>Normal weekend = home + other<br>Normal weekday holiday = home + other<br>Normal weekday during summer or winter break = home + work + other + p_w*δ*work_CW<br><br>School closure weekday = (1-d)*(home + work + other) + p_w*δ*work_CW<br>School closure weekend = (1-d)*(home + other)<br>School closure weekday holiday = (1-d)*(home + other)<br>School closure during summer or winter break = (1-d)*(home + work + other) + p_w*δ*work_CW

[a]We estimated the transmission rate of COVID-19 in the Austin-Round Rock MSA before and after the March 24th *Stay Home-Work Safe* order using least-squares fitting, which compares the predicted and observed numbers of daily hospitalizations (i.e., heads in beds) for the Austin-Round Rock MSA. We assume that: (i) the epidemic starts with 5 pre-symptomatic cases with an initial transmission rate of β, (ii) the transmission rate decreases when school closures are enacted on March 19, 2020 (by an amount determined by our pre-set contact matrices), (iii) the transmission rate decreases further by an amount d on March 25th following the *Stay Home-Work Safe* order.
[b]The methodology to estimate β and d was used with simulations initiated on each day from February 15, 2020 to March 15, 2020, and we selected the start date as the one that resulted in the smallest normalized sum of squared errors $S(\beta, d)$.

Table S4. Model parameters. Values given as five-element vectors are age-stratified with values corresponding to 0-4, 5-17, 18-49, 50-64, 65+ year age groups, respectively.

**Parameters**	**Best guess - values**	**Source**
R_0	4.1	Estimated from β
δ: doubling time	2.5 days	Estimated from β
β: transmission rate	0.053	Fitted[a] to hospitalizations data
γ^A: recovery rate on asymptomatic compartment	Equal to γ^Y	
γ^Y: recovery rate on symptomatic non-treated compartment	$\frac{1}{\gamma^Y} \sim \mathit{Triangular}(3.0{,}4.0{,}5.0)$	He et al.[11]
τ: symptomatic proportion (%)	57	Gudbjartsson et al.[15]
σ: exposed rate	$\frac{1}{\sigma} \sim \mathit{Triangular}(1.9{,}2.9{,}3.9)$	Based on incubation[14] and pre-symptomatic periods
ρ^A: pre-asymptomatic rate	Equal to ρ^Y	
ρ^Y: pre-symptomatic rate	$\frac{1}{\rho^Y} = 2.3$	He et al.[11]
P: proportion of pre-symptomatic transmission (%)	44	He et al.[11]

© 2020 Pasco RF et al. *JAMA Network Open.*

ω^P: relative infectiousness of pre-symptomatic individuals	$\omega^P = \frac{P}{1-P} \frac{\tau\omega^Y[YHR/\eta+(1-YHR)/\gamma^Y]+(1-\tau)\omega^A/\gamma^A}{\tau\omega^Y/\rho^Y+(1-\tau)\omega^A/\rho^A}$<br>$\omega^{PY} = \omega^P\omega^Y, \omega^{PA} = \omega^P\omega^A$	
ω^A: relative infectiousness of infectious individuals in compartment I^A	2/3	He et al.[12]
IFR: infected fatality ratio, age specific (%)	Overall: [0.0016, 0.0049, 0.084, 1.000, 3.371]<br>Low risk: [0.000837, 0.00194, 0.0299, 0.216, 0.5459]<br>High risk: [0.0100, 0.0233, 0.359, 2.59, 6.55]	Age adjusted from Verity et al.[19]
YFR: symptomatic fatality ratio, age specific (%)	Overall: [0.00281, 0.00868, 0.0444, 0.184, 1.75, 5.91]<br>Low risk: [0.00147, 0.00340, 0.0525, 0.379, 0.958]<br>High risk: [0.0176, 0.0408, 0.630, 4.55, 11.5]	$YFR = \frac{IFR}{\tau}$
h: high-risk proportion, age specific (%)	[8.2825, 14.1121, 16.5298, 32.9912, 47.0568]	Estimated using 2015-2016 Behavioral Risk Factor Surveillance System (BRFSS) data with multilevel regression and poststratification using CDC's list of conditions that may increase the risk of serious complications from influenza[20–22]
rr: relative risk for high risk people compared to low risk in their age group	6 for hospitalizations<br>12 for deaths	Stokes et al.[23]
School calendars	Austin Independent School District calendar (2019-2020, 2020-2021)[24]	

© 2020 Pasco RF et al. *JAMA Network Open.*

Table S5. Hospitalizations parameters

Parameters	Value	Source
γ^H: recovery rate in hospitalized compartment	0.0912	10.97 day-average from admission to discharge (Austin admissions and discharge data[16,17])
YHR: symptomatic case hospitalization rate (%)	Overall: [0.0702, 0.0702, 1.49, 5.88, 16.3, 25.5]<br>Low risk: [0.0496, 0.0411, 2.59, 6.16, 7.62]<br>High risk: [0.298, 0.247, 15.6, 37.0, 45.7]	Age adjusted from Verity et al.[19]
π: rate of symptomatic individuals who go to hospital, age-specific	$\pi = \frac{\gamma^Y \cdot YHR}{\eta + (\gamma^Y - \eta)YHR}$	
η: rate from symptom onset to hospitalized	0.1695	5.9 day average from symptom onset to hospital admission Tindale et al.[25]
μ: rate from hospitalized to death	0.128	7.8 day-average from admission to discharge (Austin admissions and discharge data[16,17])
HFR: hospitalized fatality ratio, age specific (%)	Overall: [3.21, 9.43, 2.36, 8.18, 18.5]<br>Low risk: [2.96, 8.26, 2.02, 6.15, 12.6]<br>High risk: [5.92, 16.5, 4.05, 12.3, 25.1]	$HFR = \frac{YFR}{YHR}$
ν: death rate on hospitalized individuals, age specific	$\nu = \frac{\gamma^H HFR}{\mu + (\gamma^H - \mu)HFR}$	

© 2020 Pasco RF et al. *JAMA Network Open.*

2.3. Model modification to incorporate construction workforce

We assume there are currently 50,000 construction workers in the Austin-Round Rock MSA, all in the 18-49 year-old age group. The proportion of construction workers at high-risk of complications from COVID-19 is the same as the overall 18-49y age group in the Austin-Round Rock MSA.

We extended our *US COVID-19 Pandemic Model* to include a separate population subgroup representing construction workers. We moved 50,000 individuals from the *regular* 18-49y low risk and high risk compartments into the corresponding *construction* compartments. We also extended the contact matrices[1] governing transmission between age groups to allow us to manipulate the number of construction workers and intensity of their contacts separately from the rest of the workforce. Initially, we set their contact rates equal to those of the entire 18-49y population, except that we assume that all work contacts take place within the subgroup of construction workers. Social distancing measures reduce *home, work* and *other* contacts for non-construction workers and *home* and *other* contacts for construction workers. Tables S6-S9 give the original contact matrices and Tables S10-S13 give the updated contact matrices assuming 50,000 construction workers.

Let $C(X)_{i,j}$ denote the average daily number of contacts that a person in group i has with people in group $j$at location X. Let w denote the proportion of construction workers in the 18-49y group.
For each age group i the new work (W) contact matrix between groups other than construction workers is unchanged:

$$C'(W)_{i,j} = C(W)_{i,j} \text{ for } j \neq Construction$$

$$C'(W)_{i,Construction} = 0$$

Construction workers only have contacts among themselves at work so:

$$C'(W)_{Construction,Construction} = \sum_j C(W)_{18- \quad ,j}$$

$$C'(W)_{Construction,j} = 0 \text{ for other groups } j$$

For contacts at home, school and other locations we assume that construction workers have the same contact patterns as any other 18-49 years old individual. Then at those locations (X) the contacts a person has with individuals in the 18-49y group is simply split between the existing 18-49y column and the new construction column:

$$C'(X)_{i,18-49} = C(X)_{i,18-49} * (1 - w)$$

$$C'(X)_{i,Construction} = C(X)_{i,18-49} * w$$

$$C'(X)_{Construction,j} = C'(X)_{18-4 \ ,j}$$

© 2020 Pasco RF et al. *JAMA Network Open.*

2.4. Original 5-age groups contact matrices

Age-specific contact rates were estimated by adjusting contact matrices published by Prem et al.[13]

Table S6. Home contact matrix. Daily number contacts by age group at home.

	**0-4y**	**5-17y**	**18-49y**	**50-64y**	**65y+**
**0-4y**	0.5	0.9	2.0	0.1	0.0
**5-17y**	0.2	1.7	1.9	0.2	0.0
**18-49y**	0.2	0.9	1.7	0.2	0.0
**50-64y**	0.2	0.7	1.2	1.0	0.1
**65y+**	0.1	0.7	1.0	0.3	0.6

Table S7. School contact matrix. Daily number contacts by age group at school.

	**0-4y**	**5-17y**	**18-49y**	**50-64y**	**65y+**
**0-4y**	1.0	0.5	0.4	0.1	0.0
**5-17y**	0.2	3.7	0.9	0.1	0.0
**18-49y**	0.0	0.7	0.8	0.0	0.0
**50-64y**	0.1	0.8	0.5	0.1	0.0
**65y+**	0.0	0.0	0.1	0.0	0.0

Table S8. Work contact matrix. Daily number contacts by age group at work.

	**0-4y**	**5-17y**	**18-49y**	**50-64y**	**65y+**
**0-4y**	0.0	0.0	0.0	0.0	0.0
**5-17y**	0.0	0.1	0.4	0.0	0.0
**18-49y**	0.0	0.2	4.5	0.8	0.0
**50-64y**	0.0	0.1	2.8	0.9	0.0
**65y+**	0.0	0.0	0.1	0.0	0.0

© 2020 Pasco RF et al. *JAMA Network Open.*

Table S9. Others contact matrix. Daily number contacts by age group at other locations.

	0-4y	5-17y	18-49y	50-64y	65y+
**0-4y**	0.7	0.7	1.8	0.6	0.3
**5-17y**	0.2	2.6	2.1	0.4	0.2
**18-49y**	0.1	0.7	3.3	0.6	0.2
**50-64y**	0.1	0.3	2.2	1.1	0.4
**65y+**	0.0	0.2	1.3	0.8	0.6

2.5. Updated contact matrices with separate subgroup for construction workers

Table S10. Home contact matrix. Daily number contacts by age group at home assuming 50,000 construction workers in Austin MSA.

	0-4y	5-17y	18-49y	50-64y	65y+	Construction
**0-4y**	0.5	0.9	1.9	0.1	0.0	0.1
**5-17y**	0.2	1.7	1.8	0.2	0.0	0.1
**18-49y**	0.2	0.9	1.6	0.2	0.0	0.1
**50-64y**	0.2	0.7	1.2	1.0	0.1	0.1
**65y+**	0.1	0.7	0.9	0.3	0.6	0.0
**Construction**	0.2	0.9	1.6	0.2	0.0	0.1

Table S11. School contact matrix. Daily number contacts by age group at school assuming 50,000 construction workers in Austin MSA.

	0-4y	5-17y	18-49y	50-64y	65y+	Construction
**0-4y**	1.0	0.5	0.4	0.1	0.0	0.0
**5-17y**	0.2	3.7	0.9	0.1	0.0	0.0
**18-49y**	0.0	0.7	0.8	0.0	0.0	0.0
**50-64y**	0.1	0.8	0.4	0.1	0.0	0.0
**65y+**	0.0	0.0	0.0	0.0	0.0	0.0
**Construction**	0.0	0.7	0.8	0.0	0.0	0.0

© 2020 Pasco RF et al. *JAMA Network Open.*

Table S12. Work contact matrix. Daily number contacts by age group at work assuming 50,000 construction workers in Austin MSA.

	0-4y	5-17y	18-49y	50-64y	65y+	Construction
**0-4y**	0.0	0.0	0.0	0.0	0.0	0.0
**5-17y**	0.0	0.1	0.4	0.0	0.0	0.0
**18-49y**	0.0	0.2	4.5	0.8	0.0	0.0
**50-64y**	0.0	0.1	2.8	0.9	0.0	0.0
**65y+**	0.0	0.0	0.1	0.0	0.0	0.0
**Construction**	0.0	0.0	0.0	0.0	0.0	5.5

Table S13. Others contact matrix. Daily number contacts by age group at other locations assuming 50,000 construction workers in Austin MSA.

	0-4y	5-17y	18-49y	50-64y	65y+	Construction
**0-4y**	0.7	0.7	1.7	0.6	0.3	0.1
**5-17y**	0.2	2.6	2.0	0.4	0.2	0.1
**18-49y**	0.1	0.7	3.1	0.6	0.2	0.2
**50-64y**	0.1	0.3	2.1	1.1	0.4	0.1
**65y+**	0.0	0.2	1.2	0.8	0.6	0.1
**Construction**	0.1	0.7	3.1	0.6	0.2	0.2

© 2020 Pasco RF et al. *JAMA Network Open.*

eAppendix 3. Estimation of Secondary Infections at Work and Elsewhere

We assume that, without construction work, the community-wide reproduction number is below one. Basic epidemiology tells us that if the average number of secondary infections from infected construction workers to other construction workers exceeds one, then the community-wide reproduction number will also rise above one. We use this approach to determine the level of worksite transmission risk above which construction work undermines community mitigation (i.e., raises R_0 above one).

Let R_c denote the average number of secondary infections from an infected construction worker. We decompose this quantity into $R_c = R_w + R_e$ where R_w and R_e represent the number of infections occurring at work and other locations, respectively. Our model assumes that construction workers only have contacts with other workers while at work, so that R_w represents infections among workers. In general, $1/R_w$ indicates the proportional change in transmission risk on construction sites that would raise or lower R_w to one.

Using our estimates for the population-wide reproduction number of COVID-19 in Austin (see eAppendix 2.2) and published estimates for workplace and other contact patterns (see eAppendix 2.4), we estimate R_w for the Austin area construction workforce, as follows.

- We assume that, in the absence of COVID-19 mitigation measures, the basic reproduction number for construction workers is the same as the population-wide basic reproduction number ($R_c = R_0$) and that the average number of workplace secondary infections for construction workers (R_w) is equal to the average number of secondary infections at the average workplace for the population as a whole.

- Let C_h, C_w, C_s and C_o denote the contact matrices governing interactions at home, work, school and other locations, provided in ref.[1]. We then estimate the typical weekday and weekend contact matrices as:

  $$C_{weekday} = C_h + C_w + C_s + C_o \text{ and } C_{weekend} = C_h + C_o.$$

  and the average weekly contact matrix as:

  $$C = \frac{5}{7}C_{weekday} + \frac{2}{7}C_{weekend}.$$

  Let n be vector giving the population proportions for each age group and e be the vector of ones of the same dimension as n. Then the average number of weekly contacts $\bar{c}$ in the population is given by

  $$\bar{c} = [C \cdot e]^T \cdot n.$$

  Similarly the average number of weekly workplace contacts is given by

  $$\bar{c}_w = [5/7 C_w \cdot e]^T \cdot n.$$

- Let λ be a constant proportional to the transmission rate of the virus, such that $R_0 = \lambda \cdot \bar{c}$ and $R_w = \lambda \cdot \bar{c}_w$. This implies that $R_w = R_0 \cdot \bar{c}_w / \bar{c}$.

- Given our estimate of $R_0 = 4.14$ for Austin in March 2020 (Table S4), we thereby estimate that the average number of workplace secondary infections for construction workers is $R_w = 0.88$. Given that $1/R_w = 1.136$, we estimate that a 14% increase in workplace transmission risks relative to average workplace risk would enable sustained transmission among the construction workforce and undermine community wide social distancing efforts.

© 2020 Pasco RF et al. *JAMA Network Open.*

eAppendix 4. Additional Simulation Results

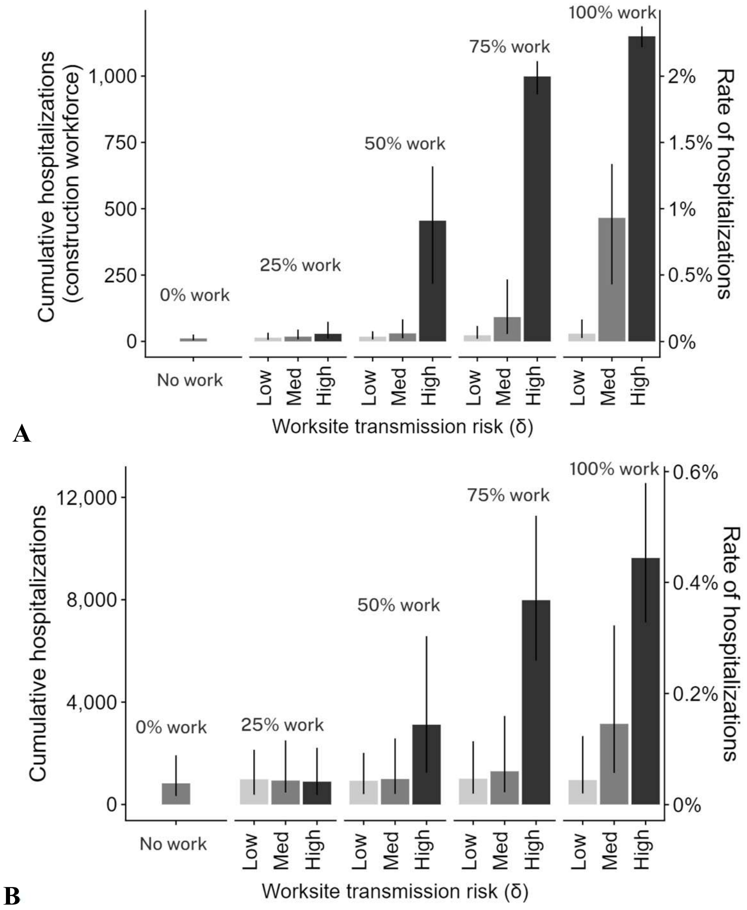

Figure S4. Projected hospitalizations for (A) construction workers and (B) the entire Austin area population through August of 2020 under different workforce scenarios. Bars and error lines indicate 25th percentile, median and 75th percentile across 500 stochastic simulations. Shading indicates level of risk of transmission at construction work sites: light gray is half the risk of a typical workplace; medium gray is typical workplace risk; dark gray is twice the risk of a typical workplace. Left axes indicate the count of hospitalizations; right axes indicate the proportion hospitalized. The transmission rate was estimated by fitting the model to observed hospitalizations in Austin up to May 3, 2020; all parameters are provided in eAppendix 2.

Table S14. Projected relative risk of COVID-19 hospitalization among construction workers under all construction work scenarios. A value greater than 1 indicates that construction workers are at higher risk of hospitalization due to COVID-19 infection than members of the general population in the same age group.

	**Worksite transmission risk**		
**Percent of workforce**	**50% risk**	**Baseline risk**	**200% risk**
0%	0.70	0.70	0.69
25%	0.76	1.01	1.73
50%	1.00	1.59	8.74
75%	1.22	3.95	7.49
100%	1.61	8.65	7.11

4.1 Sensitivity analysis: Varying the size of the construction workforce, the worksite transmission risk and proportion of construction workers that are high risk for severe COVID-19

In our main results, we assume that the high risk proportion among construction workers is the same as the general 18-49y population. Thus, the estimated excess risk of hospitalizations among construction workers stems solely from increased exposure at work. Given that the construction workforce may experience higher rates of high risk conditions[37,38] stemming from overlapping socioeconomic, racial, occupational and health vulnerabilities, we varied the high risk proportion for the construction workforce. Specifically, we held the overall high risk proportion constant (for the population as a whole) and adjusted the ratio between the construction high risk proportion and the general 18-49y high risk proportion. We considered eight different ratios ranging from 0.8 (high risk proportion 20% lower in construction workers) to 2.0 (proportion double in construction workers).

Let H_w and H_{18-49} denote the proportion of high-risk individuals among construction workers and among 18-49y in the general population, respectively. We varied the number of high-risk construction workers and 18-49y such that:

$$\frac{H_w}{H_{18-49}} = x, \text{ for } x \in \{0.8, 0.9, 1.0, 1.1, 1.25, 1.5, 1.75, 2.0\}.$$

The primary analysis presented in the manuscript assumes equal high risk proportions in the two groups and corresponds to $\frac{H_w}{H_{18-49}} = 1.0$. For each of these ratios, the corresponding numbers and proportions of high-risk construction workers and other adults in the 18-49 group are given in table S14.

Table S15. Numbers and proportions of high-risk individuals among construction workers and non-construction workers in the 18-49y age group for the eight different risk scenarios. The ratio of high risk proportions, given as column headings, equals the proportion in the second row divided by the proportion in the fourth row.

		Relative Proportion of high-risk individuals							
**Group**	**Metric**	**0.8**	**0.9**	**1**	**1.1**	**1.25**	**1.5**	**1.75**	**2**
**Construction Workers**	**Count**	5,883	6,587	7,285	7,976	9,002	10,679	12,318	13,921
	**Proportion (%)**	11.8	13.2	14.6	16.0	18.0	21.4	24.6	27.8
**18-49**	**Count**	150,326	149,622	148,924	148,233	147,207	145,530	143,891	142,288
	**Proportion (%)**	14.7	14.6	14.6	14.5	14.4	14.2	14.1	13.9

Figure S5 compares the estimated relative risk of hospitalizations for construction workers through August 17, 2020 across the eight different scenarios. As in Figure 3, we calculated the ratio of hospitalization rate among construction workers and the hospitalization rate among other 18-49y.

© 2020 Pasco RF et al. *JAMA Network Open.*

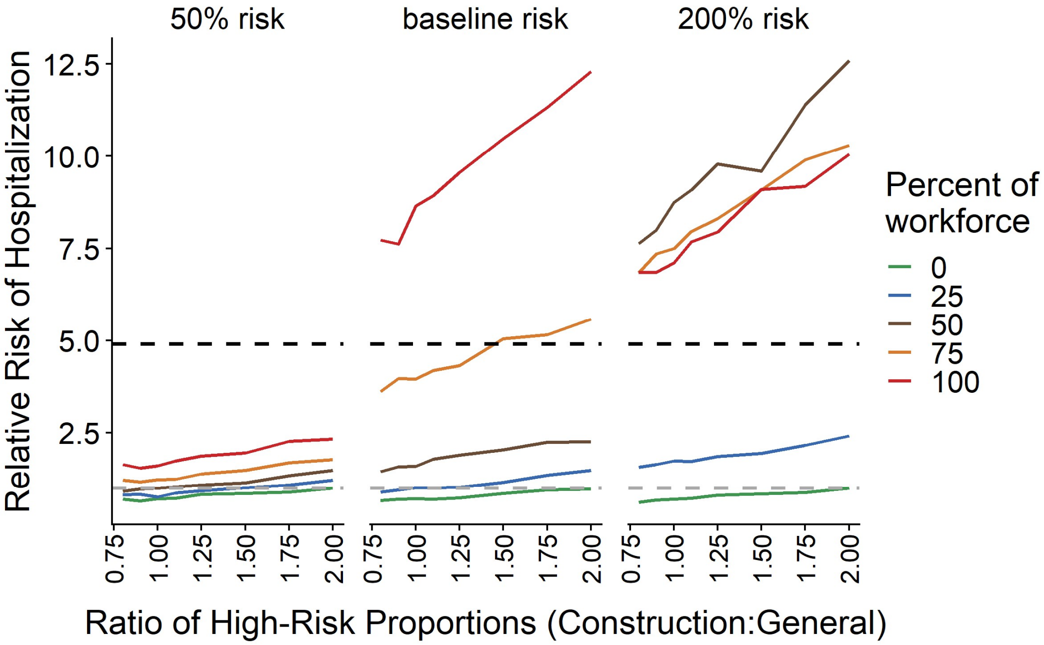

Figure S5. Projected relative risk of hospitalization among construction workers by August 17, 2020 depending on the size of the workforce (colors), worksite transmission risks (columns) and prevalence of high risk conditions among construction workers (x-axis). The y-axis indicates the relative risk of hospitalizations among construction workers versus other 18-49 year olds. The x-axes indicate the ratio between the proportion of construction workers with high risk conditions and the proportion of other 18-49 year olds with high risk conditions. Relative risks are estimated as medians across 500 simulations for each scenario. For each simulation, we calculate the ratio between the cumulative proportion of construction workers and 18-49y hospitalized by August 17, 2020. The grey dashed line at a value of one indicates that construction workers have the same risk of hospitalization as the general population; the black dashed line at 4.9 is the value estimated from Austin area hospitalization data through August 20, 2020.

eReferences

1. Prem K, Cook AR, Jit M. Projecting social contact matrices in 152 countries using contact surveys and demographic data. *PLoS Comput Biol*. 2017;13(9):e1005697. doi:10.1371/journal.pcbi.1005697

2. Keeling MJ, Rohani P. *Modeling Infectious Diseases in Humans and Animals*. Princeton University Press; 2011. https://play.google.com/store/books/details?id=LxzILSuKDhUC

3. Gillespie DT. Approximate accelerated stochastic simulation of chemically reacting systems. *J Chem Phys*. 2001;115(4):1716-1733. doi:10.1063/1.1378322

4. US Census Bureau. American Community Survey (ACS). Accessed November 19, 2019. https://www.census.gov/programs-surveys/acs

5. 500 Cities Project: Local data for better health | Home page | CDC. Published December 5, 2019. Accessed March 19, 2020. https://www.cdc.gov/500cities/index.htm

6. for Disease Control C, Prevention, Others. HIV surveillance report. 2016; 28. *URL: http://www cdc gov/hiv/library/reports/hiv-surveil-lance html Published November*. Published online 2017.

7. Morgan OW, Bramley A, Fowlkes A, et al. Morbid obesity as a risk factor for hospitalization and death due to 2009 pandemic influenza A(H1N1) disease. *PLoS One*. 2010;5(3):e9694. doi:10.1371/journal.pone.0009694

8. Sturm R, Hattori A. Morbid obesity rates continue to rise rapidly in the United States. *Int J Obes* . 2013;37(6):889-891. doi:10.1038/ijo.2012.159

9. Estimating the Number of Pregnant Women in a Geographic Area from CDC Division of Reproductive Health. https://www.cdc.gov/reproductivehealth/emergency/pdfs/PregnacyEstimatoBrochure508.pdf

10. Workforce overview. Austin Chamber of Commerce. Accessed March 30, 2020. https://www.austinchamber.com/economic-development/austin-profile/workforce/overview

11. He X, Lau EHY, Wu P, et al. Temporal dynamics in viral shedding and transmissibility of COVID-19. *Nat Med*. Published online April 15, 2020. doi:10.1038/s41591-020-0869-5

12. He D, Zhao S, Lin Q, et al. The relative transmissibility of asymptomatic COVID-19 infections among close contacts. *Int J Infect Dis*. 2020;94:145-147. doi:10.1016/j.ijid.2020.04.034

13. Prem K, Cook AR, Jit M. Projecting social contact matrices in 152 countries using contact surveys and demographic data. *PLoS Comput Biol*. 2017;13(9):e1005697. doi:10.1371/journal.pcbi.1005697

14. Zhang J, Litvinova M, Wang W, et al. Evolving epidemiology and transmission dynamics of coronavirus disease 2019 outside Hubei province, China: a descriptive and modelling study. *Lancet Infect Dis*. Published online April 2, 2020. doi:10.1016/S1473-3099(20)30230-9

15. Gudbjartsson DF, Helgason A, Jonsson H, et al. Spread of SARS-CoV-2 in the Icelandic Population. *N Engl J Med*. Published online April 14, 2020. doi:10.1056/NEJMoa2006100

16. Richardson S, Hirsch JS, Narasimhan M, et al. Presenting Characteristics, Comorbidities, and Outcomes Among 5700 Patients Hospitalized With COVID-19 in the New York City Area. *JAMA*. Published online April 22, 2020. doi:10.1001/jama.2020.6775

17. Lewnard JA, Liu VX, Jackson ML, et al. Incidence, clinical outcomes, and transmission dynamics of hospitalized 2019 coronavirus disease among 9,596,321 individuals residing in California and Washington, United States: a prospective cohort study. *Epidemiology*. Published online April 16, 2020. doi:10.1101/2020.04.12.20062943

18. minimize(method='trust-constr') — SciPy v1.4.1 Reference Guide. Accessed April 19, 2020.

© 2020 Pasco RF et al. *JAMA Network Open.*

https://docs.scipy.org/doc/scipy/reference/optimize.minimize-trustconstr.html

19. Verity R, Okell LC, Dorigatti I, et al. Estimates of the severity of coronavirus disease 2019: a model-based analysis. *Lancet Infect Dis*. Published online March 30, 2020. doi:10.1016/S1473-3099(20)30243-7

20. CDC. People at High Risk of Flu. Centers for Disease Control and Prevention. Published November 1, 2019. Accessed March 26, 2020. https://www.cdc.gov/flu/highrisk/index.htm

21. CDC - BRFSS. Published November 5, 2019. Accessed March 26, 2020. https://www.cdc.gov/brfss/index.html

22. Zhang X, Holt JB, Lu H, et al. Multilevel regression and poststratification for small-area estimation of population health outcomes: a case study of chronic obstructive pulmonary disease prevalence using the behavioral risk factor surveillance system. *Am J Epidemiol*. 2014;179(8):1025-1033. doi:10.1093/aje/kwu018

23. Stokes EK, Zambrano LD, Anderson KN, et al. Coronavirus Disease 2019 Case Surveillance - United States, January 22-May 30, 2020. *MMWR Morb Mortal Wkly Rep*. 2020;69(24):759-765. doi:10.15585/mmwr.mm6924e2

24. Calendar of Events. Austin ISD. Accessed March 26, 2020. https://www.austinisd.org/calendar

25. Tindale L, Coombe M, Stockdale JE, et al. Transmission interval estimates suggest pre-symptomatic spread of COVID-19. *Epidemiology*. Published online March 6, 2020. doi:10.1101/2020.03.03.20029983

26. Health Outcomes | 500 Cities. Published April 25, 2019. Accessed March 28, 2020. https://www.cdc.gov/500cities/definitions/health-outcomes.htm

27. Part One: Who Lives with Chronic Conditions. Pew Research Center: Internet, Science & Tech. Published November 26, 2013. Accessed November 23, 2019. https://www.pewresearch.org/internet/2013/11/26/part-one-who-lives-with-chronic-conditions/

28. Miller GF, Coffield E, Leroy Z, Wallin R. Prevalence and Costs of Five Chronic Conditions in Children. *J Sch Nurs*. 2016;32(5):357-364. doi:10.1177/1059840516641190

29. Cancer Facts & Figures 2014. Accessed March 30, 2020. https://www.cancer.org/research/cancer-facts-statistics/all-cancer-facts-figures/cancer-facts-figures-2014.html

30. Hales CM, Fryar CD, Carroll MD, Freedman DS, Ogden CL. Trends in Obesity and Severe Obesity Prevalence in US Youth and Adults by Sex and Age, 2007-2008 to 2015-2016. *JAMA*. 2018;319(16):1723-1725. doi:10.1001/jama.2018.3060

31. Zimmerman RK, Lauderdale DS, Tan SM, Wagener DK. Prevalence of high-risk indications for influenza vaccine varies by age, race, and income. *Vaccine*. 2010;28(39):6470-6477. doi:10.1016/j.vaccine.2010.07.037

32. Martin JA, Hamilton BE, Osterman MJK, Driscoll AK, Drake P. Births: Final Data for 2017. *Natl Vital Stat Rep*. 2018;67(8):1-50. https://www.ncbi.nlm.nih.gov/pubmed/30707672

33. Jatlaoui TC, Boutot ME, Mandel MG, et al. Abortion Surveillance - United States, 2015. *MMWR Surveill Summ*. 2018;67(13):1-45. doi:10.15585/mmwr.ss6713a1

34. Ventura SJ, Curtin SC, Abma JC, Henshaw SK. Estimated pregnancy rates and rates of pregnancy outcomes for the United States, 1990-2008. *Natl Vital Stat Rep*. 2012;60(7):1-21. https://www.ncbi.nlm.nih.gov/pubmed/22970648

35. Killerby ME, Link-Gelles R, Haight SC, et al. Characteristics Associated with Hospitalization Among Patients with COVID-19 - Metropolitan Atlanta, Georgia, March-April 2020. *MMWR Morb Mortal Wkly Rep*. 2020;69(25):790-794. doi:10.15585/mmwr.mm6925e1

36. Hamer M, Kivimäki M, Gale CR, Batty GD. Lifestyle risk factors, inflammatory mechanisms, and COVID-19

© 2020 Pasco RF et al. *JAMA Network Open.*

hospitalization: A community-based cohort study of 387,109 adults in UK. *Brain Behav Immun*. 2020;87:184-187. doi:10.1016/j.bbi.2020.05.059

37. CDC. Coronavirus Disease 2019 (COVID-19). Published August 14, 2020. Accessed September 10, 2020. https://www.cdc.gov/coronavirus/2019-ncov/need-extra-precautions/people-with-medical-conditions.html?CDC_AA_refVal=https%3A%2F%2Fwww.cdc.gov%2Fcoronavirus%2F2019-ncov%2Fneed-extra-precautions%2Fgroups-at-higher-risk.html

38. CPWR Chart Book (6th edition): Health Indicators and Services - Health Risk Factors and Chronic Illnesses among Construction Workers. Accessed September 10, 2020. https://www.cpwr.com/research/data-center/the-construction-chart-book/chart-book-6th-edition-health-indicators-and-services-health-risk-factors-and-chronic-illnesses-among-construction-workers/

© 2020 Pasco RF et al. *JAMA Network Open.*
